# Advancing the use of evidence in bouillon fortification policy discussions: Burkina Faso, Nigeria, and Senegal

**DOI:** 10.1111/nyas.70006

**Published:** 2025-08-23

**Authors:** Ann Tarini, Jérome W. Somé, Maguette Beye, Faith Ishaya, Karim Koudougou, Augustine Okoruwa, Stephen A. Vosti

**Affiliations:** ^1^ Consultant Laval Québec Canada; ^2^ Institut de Recherche en Sciences de la Santé Centre National de Recherche Scientifique et Technologique Ouagadougou Burkina Faso; ^3^ Helen Keller International Dakar Senegal; ^4^ Helen Keller International Abuja Nigeria; ^5^ Helen Keller International Ouagadougou Burkina Faso; ^6^ Department of Agricultural and Resource Economics University of California Davis California USA; ^7^ Institute for Global Nutrition, Department of Nutrition University of California Davis California USA

**Keywords:** evidence‐based decision‐making, fortified bouillon, micronutrient policy, policy engagement, West Africa

## Abstract

Policy changes require stakeholder buy‐in, therefore, the timely delivery of tailored evidence provided to all stakeholders involved in policy discussions is important. This paper reports the evidence generation and delivery processes undertaken in Burkina Faso, Nigeria, and Senegal in support of bouillon fortification discussions. We identified stakeholder‐specific evidence needs, tapped existing data and new data to generate that evidence, and packaged and delivered it to stakeholders. Evidence needs included the levels of micronutrient inadequacy (with/without existing and other hypothetical fortification programs), the potential contributions of bouillon fortification to reduce micronutrient inadequacy and (for some micronutrients) child mortality, the cost and cost‐effectiveness of bouillon fortification programs, and the contribution of bouillon to total sodium intake. Evidence on technical and commercial issues was also required. Stakeholder‐specific understanding and ownership of evidence was essential; achieving both required continual interaction and trust. New bouillon evidence delivery channels were developed in each country and linked to existing decision‐making bodies. A shared vocabulary and understanding of issues and evidence, and the continual innovative redelivery of evidence, were critical to success; persistence and innovations in evidence delivery paid dividends. Concrete policy changes regarding bouillon fortification were secured in Nigeria; policy discussions continue in Burkina Faso and Senegal.

## Graphical abstract

Nutrition policy landscapes are comprised of a large number of stakeholders and vary by country depending on the policy issues being discussed. Launching a bouillon fortification program required mapping and strategically engaging with selected entities and individuals in these landscapes, discovering their evidence needs regarding specific questions, generating the needed evidence, and devising ways to deliver compelling evidence in innovative and timely ways.

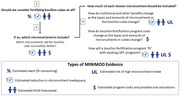

## INTRODUCTION

Inadequacy of key micronutrients remains a problem in many lower‐ and middle‐income countries (LMICs), especially for young children and women of reproductive age (WRA).^1^, ^2^, ^3^ Large‐scale food fortification (LSFF) programs are commonly recommended to reduce micronutrient inadequacy.^4^, ^5^, ^6^, ^7^ However, the potential contributions of LSFF programs to reducing inadequacies are sometimes limited.^8^, ^9^ This situation can be attributed, in part, to programs not being designed based on context‐specific evidence, which can result in the selection of food vehicles that are not widely consumed in a fortifiable form by at‐risk individuals,^10^ fortification standards that are not aligned with populations’ dietary habits and (hence) nutritional needs,^11^ and/or the inability to effectively monitor programs and make necessary adjustments. For instance, surveys conducted in 16 LMICs showed that despite the potential of some food vehicles to reach vulnerable populations, LSFF programs fall short due to improper assumptions regarding program coverage and/or low levels of compliance with mandatory fortification standards.^9^ Some of these shortcomings may be due to the absence of key evidence during the LSFF food vehicle selection and program design phases, or the failure to deliver existing evidence to key stakeholders in timely ways throughout the implementation process, hence, more and better‐delivered evidence might help avoid LSFF program shortfalls, especially if provided during the early stages of program development.

**FIGURE 1:**
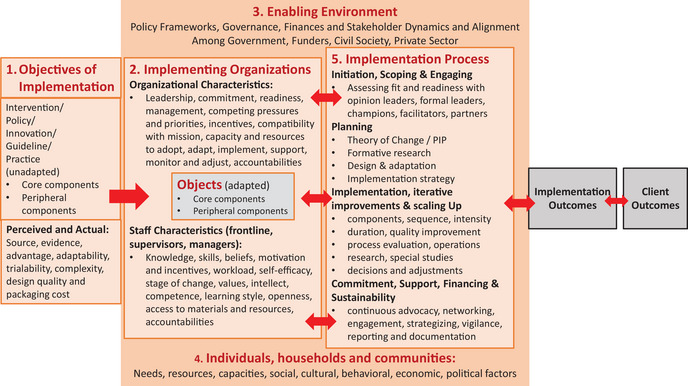
Five domains for enhancing implementation and impact of nutrition intervention programs. Figure adapted from Ref. ^64^

The broad and multisectoral literature on implementation science research provides high‐level frameworks and methods for meeting these challenges.^12^, ^13^, ^14^ ^,^ a For example, Jacobson et al. set out a theory of knowledge translation (knowledge utilization) for decision‐making.^15^ Their framework focused on five domains to consider when setting up a system for the use of evidence, namely: (1) the user group, (2) the issue, (3) the research, (4) the knowledge translation relationship, and (5) dissemination strategies. Tumilowicz et al. further identified five domains that research could support to enhance the implementation and impact of nutrition intervention programs.^16^ These domains are: (1) the intervention itself; (2) the implementing organization(s); (3) the enabling environment of policies and stakeholders; (4) the target individuals, households, and communities; (5) and the strategies used throughout the implementation process. Figure 1 summarizes these domains and provides some details regarding subissues associated with each.

Shroff et al. identified five key factors for the success of incorporating evidence in public health policymaking based on work in five LMICs: (1) engaged policymakers; (2) research issues of mutual interest for researchers and users; (3) accessible, methodologically sound research; (4) strong researcher–policymaker relationships; (5) clear outcomes and broad dissemination.^17^ Recognizing the influence of individual leaders on institutional fragility (e.g., uncertain funding, frequent changes in mandates, and high staff turnover) in some settings, they also suggested adding leadership as a sixth factor.

**FIGURE 2:**
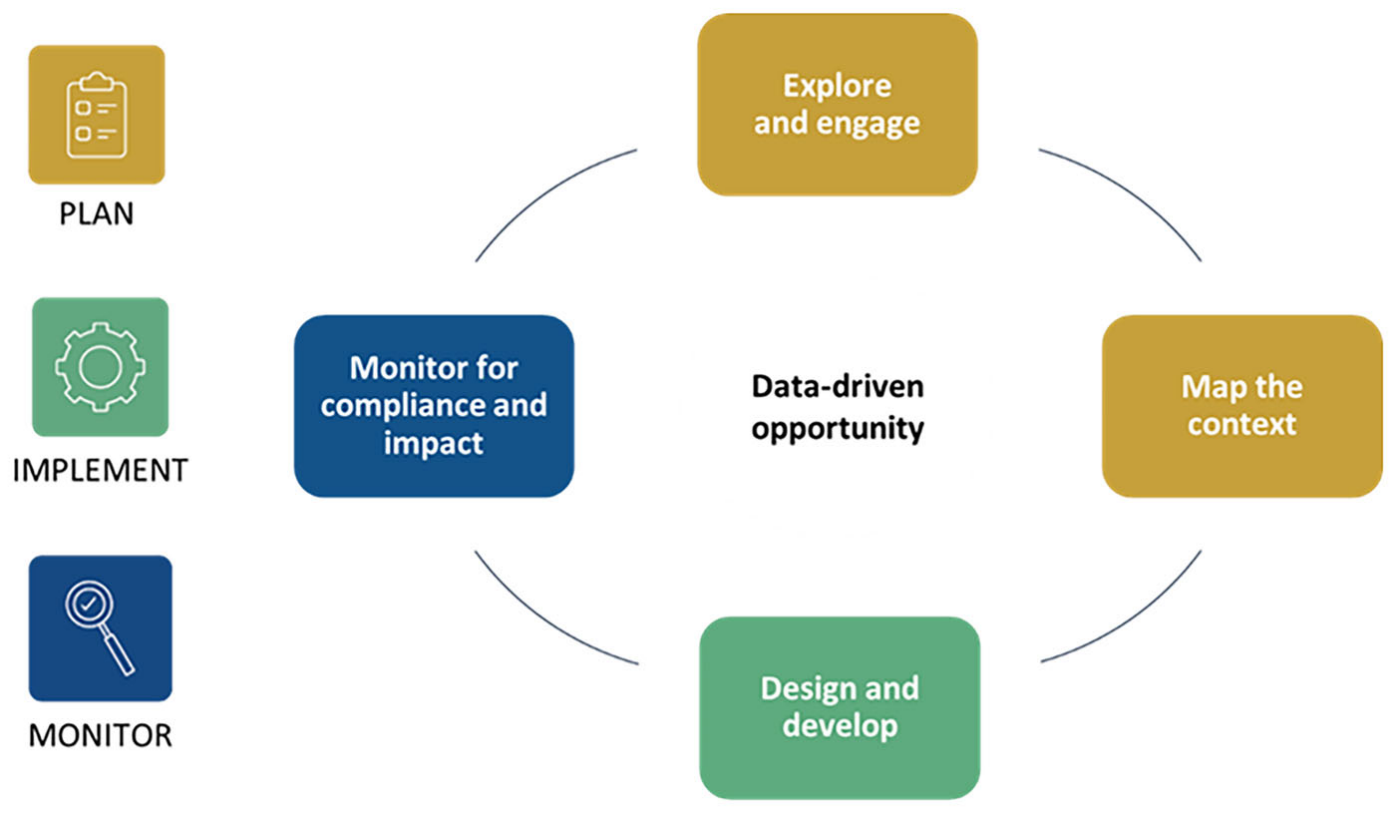
Strategic phases of food fortification program design, implementation, and management. *Source*: Food Fortification Initiative.^20^

More directly related to designing, implementing, and managing LSFF programs, literature exists regarding how to choose food/condiment vehicles for delivering needed micronutrients (e.g., Allen et al.^18^), the pathways for designing and managing LSFF programs,^19^ and how to manage all phases of such projects^20^ (see Figure 2 for an overview).

Finally, the Society for Implementation Science in Nutrition^21^ was established to refine materials and methods developed by implementation scientists working in other domains, and apply them to nutrition problems and policies, with a particular focus on LMICs.

These literatures provide a comprehensive, high‐level point of departure for the evidence generation and delivery activities associated with the multifortified bouillon project examined in this paper, but also emphasizes the need to identify and address country‐ and delivery vehicle–specific issues.^13^, ^14^, ^20^ Therefore, our contributions to the existing literature are derived from applying these general frameworks to the case of hypothetical multifortified bouillon cubes in three country contexts—Burkina Faso, Nigeria, and Senegal. This was done as part of the project “Accelerating reductions in micronutrient deficiencies in West Africa through fortified bouillon,” which aimed at addressing gaps in evidence on the effectiveness and cost‐effectiveness of fortifying bouillon with multiple micronutrients to improve dietary adequacy and reduce nutrient deficiencies.^b^ In this context, sets of evidence (alongside other evidence produced by other groups) on the nutritional benefits and costs of adding five key micronutrients, particularly for children and WRA, were produced for each country and integrated into policy discussions.

Applying existing general frameworks required several refinements because: (1) although some producers were voluntarily fortifying small amounts of single micronutrients, bouillon was a new vehicle for delivering multiple micronutrients; (2) bouillon cubes are mainly comprised of salt and, therefore, in most cases, already conveyed iodine to consumers, but high sodium intake was a concern in all project countries; (3) from the perspective of bouillon fortification, a new industry, a new set of producers, new food technology challenges, and new input value chains were involved; (4) in some cases, very substantial proportions of bouillon were imported; and (5) the policymaking, regulatory, and so on processes were different across the three countries included in this project.

In these new and narrow contexts, our contributions include methods for: (1) identifying stakeholders and especially stakeholder‐specific evidence needs^c^ at the outset of the project; (2) generating the evidence to meet those stakeholder‐specific needs; and (3) distilling/packaging/delivering evidence to stakeholders in timely and impactful ways. Our higher‐level contributions are the lessons learned while discovering and managing the spaces for discussion and the channels through which stakeholder‐specific, bidirectional information flowed. Our contributions primarily focus on the exploration and planning phases, where we consider potential design options for a program using a new fortification vehicle, but the evidence bases included information relevant for subsequent program implementation and management as well.

## CONTEXT CHARACTERISTICS AND METHODS

### Data and context characteristics

Global estimates suggest that micronutrient deficiencies are still a remarkable burden in sub‐Saharan Africa.^1^ However, detailed national and subnational data on deficiencies (micronutrient status) or their causes (e.g., 24‐h dietary intake data) are not generally available, or if they are available, are sometimes not used to inform food fortification policy discussions. Below, we described available information on micronutrient status, dietary intake, and micronutrient intervention program implementation in each of the selected countries at the time of this study.

In Burkina Faso, the recently published results from the Ministry of Health's (MoH) 2020 national micronutrient survey show a significant prevalence of deficiencies among children aged 6−59 months: 39% for iron deficiency, 22% for iron deficiency anemia, and 50% for vitamin A deficiency. Zinc deficiency stood at 13%, while vitamin B12 deficiency was present in 12% of children. Lastly, 2% of children showed folate deficiency (measured in either red blood cells or serum).^22^ The 2018 Fortification Assessment Coverage Tool (FACT) market survey^20^ assessed fortification levels of oil, salt, and wheat flour in eight market hubs, revealing widespread noncompliance with national fortification standards.^23^ Results showed that many fortifiable foods at retail outlets were not fortified or fortified at amounts below national standards. Furthermore, the results of the 2020 national micronutrient survey showed that only 57% of salt samples were adequately fortified with iodine (≥ 15 ppm) and 72.4% of oil samples contained no vitamin A.^24^ LSFF program monitoring data generally are not shared outside of the government. Finally, Household Consumption and Expenditure Surveys (HCES) are regularly conducted in the framework of the “Enquête Harmonisée sur les Conditions de Vie des Ménages” (an LSMS‐type survey), the most recent survey for which data are available was undertaken in 2018–2019.^25^ While these data have been tapped for other purposes related to human nutrition,^26^, ^27^ they had not previously been used to estimate micronutrient inadequacies or to explore programmatic options to address them.

In Senegal, data on micronutrient deficiencies available at the onset of the project were sourced from a 2010 household survey assessing iron, vitamin A, and zinc deficiencies among women aged 15−49 and children 12−59 months.^28^ Findings revealed high levels of micronutrient deficiencies: 44.7% of women and 61.5% of children were iron deficient, 66.7% of women and 39.6% of children were zinc deficient, and 18.2% of children had vitamin A deficiency. Only 12.8% of women and 11.6% of children had no deficiencies. As noted above in the case of Burkina Faso, LSFF program monitoring data generally are not shared outside of government entities of Senegal. Household Consumption and Expenditure data are regularly collected in the framework of the “Enquête Harmonisée sur les Conditions de Vie des Ménages,” the most recent database available being the 2018–2019 survey.^29^ As was the case of Burkina Faso, these data had not previously been used for nutrition‐related analyses.

Nigeria undertook a nationally representative micronutrient deficiency (based on biomarkers) and 24‐h recall dietary intake data collection in 2021; a preliminary report based primarily on market data was released in 2023.^30^ Final reports on the deficiency and dietary intake data were recently released,^31^ but the underlying data, including recipe data supporting the dietary intake survey, have not yet been released. As is the case of Burkina Faso and Senegal, Nigeria also routinely undertakes Living Standards Measurement Survey^32^ data which contain, among many other things, detailed household‐level assessments of food purchases and consumption. Several FACT surveys were undertaken in Nigeria over the past decade.^33^, ^34^ Therefore, while more was known about micronutrient intake in Nigeria, once again, these nationally representative data had not previously been used for nutrition‐related analyses.

### Existing LSFF programs

Burkina Faso and Senegal have three LSFF programs (and associated mandatory standards): iodized salt (15–20 ppm [Burkina Faso] and 20–60 ppm [Senegal]), evaluated at household level,^44^, ^46^ vegetable oil fortified with vitamin A (17.5 mg/kg of retinyl palmitate), and wheat flour fortified with iron (60 mg/kg) and folic acid (2.5 mg/kg).^d^ The process for launching LSFF in these two countries was supported by development organizations and international consultants who supported the needed analysis, developed standards and guidelines, and undertook capacity‐building activities. Partners also supported the creation of the National Alliance for Food Fortification. Once established, however, activities such as compliance monitoring and meetings of the National Alliance for Food Fortification became less rigorous and frequent.

Nigeria has a relatively long history in food/condiment fortification.^35^ The first program (iodization of salt, 50 mg/kg) was established in 1992 and continues to this day. Mandatory fortification of edible oils (vitamin A, 6 mg/kg) was established in 2000. Mandatory fortification of maize flour and wheat/semolina flour (same standard for all products: B6, 6 mg/kg; B12, 0.02 mg/kg; B9, 2.6 mg/kg; iron, 40 mg/kg; B3, 45 mg/kg; B2, 5 mg/kg; B1, 6 mg/kg; vitamin A 2 mg/kg; and zinc, 50 mg/kg) was introduced in 2010. The fortification with vitamin A of sugar (7.5 mg/kg) sold directly to consumers is also mandatory (sugar sold to bakeries, etc., need not be fortified). Discussions related to the multifortification of rice (using fortified kernel extrusion technology) are ongoing. Therefore, the institutional and legal frameworks associated with setting fortification standards, and monitoring and regulating fortification programs have been in place in Nigeria for over two decades. This collection of organizations and actors provided a ready home for discussions around the multifortification of bouillon.

In all three countries, organizations concerned about and having a mandate for addressing excess sodium intake and associated noncommunicable diseases had been established,^36^ and all played fundamental roles in highlighting the concerns regarding the salt content of bouillon and bouillon consumption, and in ensuring that evidence on sodium intake (with particular focus on the contributions of bouillon to total sodium intake) be gathered and considered by the national bouillon country working groups (CWGs).

### Activities and methods

To operationalize the co‐development and delivery of evidence in the context of the multifortified bouillon project, the team undertook the following collaborative activities.

First, we constructed nutrition policy landscape maps to identify the key actors and relationships that were relevant for discussions regarding fortified bouillon through informal exchange with local actors and collaborators well‐informed about each country's nutrition and food fortification program contexts.

Second, we developed a generic, multistakeholder framework that facilitated the: (1) identification of stakeholder groups involved in bouillon fortification program discussions, (2) identification/cultivation^e^ of the evidence needs of each group, (3) translation of evidence needs into researchable questions and the data/methods required to address them, and (4) identification of pathways for delivering new evidence back to stakeholder groups.

Third, we created entities and discussion spaces specifically for fortified bouillon and linked them to existing entities and discussion spaces associated with LSFF programs generally.

Fourth, we generated and delivered essential evidence for policy discussions, and worked with stakeholders, the Micronutrient Intervention Modeling (MINIMOD) modeling team, and other providers of evidence.

Fifth, and closely related, we identified stakeholder‐specific ways to foster understanding of, and interest and confidence in, the research tools, their underlying data, and the types of evidence that the tools could generate.

Sixth, we developed a set of best practices and lessons learned based on our own experiences and on the results of semi‐structured interviews with selected project leads and other in‐country stakeholders.^f^

Finally, we set our experiences alongside an example of the very successful Neglected Tropical Diseases (NTD) project to assess differences/similarities in approaches, evidence generation and use, and outcomes.

## RESULTS

### Nutrition policy landscape maps

While Figure 1 and the associated text are generically accurate, they beg the issues of which specific stakeholder groups (of the many that generally exist) to engage with, what pathways link them with decision‐makers, and what types of evidence each stakeholder group needs (when and in what format) to engage in discussions regarding the design and management of a multifortified bouillon program.

To help make the various stakeholder groups concrete and (more importantly) to help prioritize which stakeholder and decision‐making groups to engage with, we mapped the nutrition policy landscapes in each study country. We present them below. As one would expect, they are similar in structure, with all nutrition policy decisions being made at the level of the MoH, but the details of the nutrition policy landscape maps differed across countries and these differences helped us derive country‐specific strategies for MINIMOD policy engagement activities and objectives. For example, in Senegal, strong government leadership in nutrition prompted them to spearhead MINIMOD discussions once they grasped the basics of the tool, organizing meetings and shaping messages for other national stakeholders. In Burkina Faso, by contrast, different approaches were required due to the general lack of understanding of and interest in LSFF programs within the MoH and the partners who supported or influenced them. In this case, the research and policy engagement team faced the double challenge of advocating for greater attention to LSFF programs in general and for considering bouillon fortification in particular.

The nutrition policy landscapes identify the various governmental and other agencies involved in nutrition policy discussions, their interactions, their roles in LSFF program discussions in general, and their roles in bouillon fortification decisions in particular. These landscape maps were used to develop strategies for interacting with these groups, sometimes creating discussion spaces to achieve our objectives related to bouillon fortification. Much more elaborate nutrition landscape maps have been produced in other contexts (e.g., Kohli et al.^37^), but to our knowledge, none have focused on stakeholder‐specific evidence needs and the activities/data required to meet them.

Notably, National Fortification Alliances (NFAs) were in place in all three countries to oversee food/condiment fortification programs. However, the NFAs focused mainly on existing LSFF programs and their management, so we created entities/discussion spaces specifically for fortified bouillon and linked them to existing entities/discussion spaces associated with LSFF programs generally. More specifically, Bouillon CWGs were formed in all countries and co‐managed by Helen Keller International (HKI). These CWGs were comprised of key stakeholders and were commissioned by and reported to the NFAs. The CWGs’ core responsibilities were to gather evidence related to the nutritional benefits and costs associated with the multifortification of bouillon, and to make recommendations to the NFAs regarding bouillon fortification, including the types and amounts of fortificants to be included in premixes. The CWGs were transient and would be dissolved upon submission of their final reports to the respective NFAs.

**FIGURE 3:**
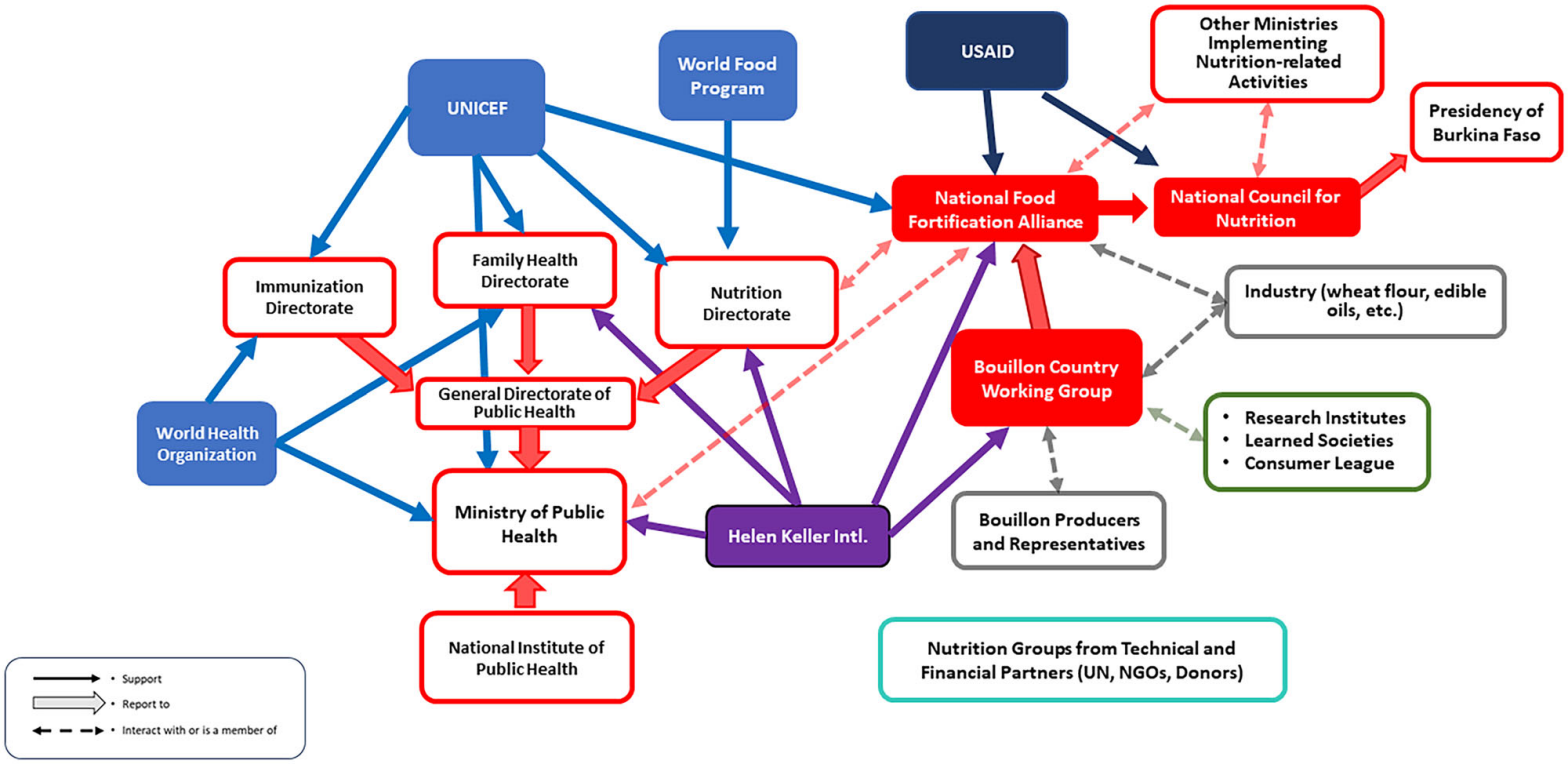
Nutrition policy landscape relevant for bouillon fortification program discussions in Burkina Faso. Red/red‐outlined figures represent national government organizations/agencies; blue figures represent UN agencies; dark blue figures represent international development agencies; purple figures represent NGOs/international NGOs; green and gray figures represent civil society and industry, respectively; and nutrition groups within technical and financial partner organizations have the potential to interact with all other organizations/agencies identified in the figure. Arrows indicate information and influence pathways. Abbreviations: NGO, nongovernmental organization; UN, United Nations; UNICEF, United Nations International Children's Emergency Fund; USAID, United States Aid for International Development.

In Burkina Faso (Figure 3), NFA is chaired by the MoH, represented by the Nutrition Directorate that is also in charge of other micronutrient intervention programs (e.g., vitamin A supplementation programs and the distribution of multiple micronutrient powders [MNPs] to children). Members of NFA are representatives of public institutions responsible for monitoring and regulation. These types of institutions include the Agence Nationale pour la Sécurité Sanitaire de l'Environnement, de l'Alimentation, du Travail et des Produits de Santé (ANSSEAT), the Direction Générale des Douanes (DGD), the Institut de Recherche en Sciences Appliquées et Technologies (IRSAT), the Institut de Recherche en Sciences de la Santé (IRSS), and the Direction de la Nutrition (DN). Civil society (e.g., the Réseau de la Société Civile pour la Nutrition [RESONUT]) and international organizations (e.g., HKI, UNICEF, WFP, WHO, Nutrition International, and Groupe de Recherche et d'Echange Technologique [GRET]) were also represented. NFA meetings occurred irregularly, often tied to externally supported projects. HKI, CRS, UNICEF, and USAID assisted in organizing and managing NFA meetings and LSFF endeavors. The Burkina Faso bouillon CWG was comprised of a subset of NFA permanent members, bouillon cube producer and importer representatives, and a representative of the cardiologist society. The most active nutrition coordination platforms included the UNICEF‐led nutrition partner group and the government‐led multisectoral platform Conseil National de Nutrition (CNaN) (as of 2025). Partner meetings convened monthly, while national‐led meetings occurred biannually, with recent frequency reductions due to structural shifts.

These platforms mainly covered health‐related nutrition matters, with fortification discussions primarily centered on home fortification with MNPs.

Despite the existence of an interconnected set of nutrition policy stakeholder groups, there were weak links in the context of Burkina Faso. For example, the Nutrition Directorate lacked sufficient expertise on LSFF programs, mainly due to staff turnover. Micronutrient policies and programs other than vitamin A supplementation or MNPs received little support from external partners. Therefore, although linkages existed, NFA and CWG discussions tended to be isolated from broader nutrition coordination bodies, hampering LSFF program visibility within the government and among development partners.

**FIGURE 4:**
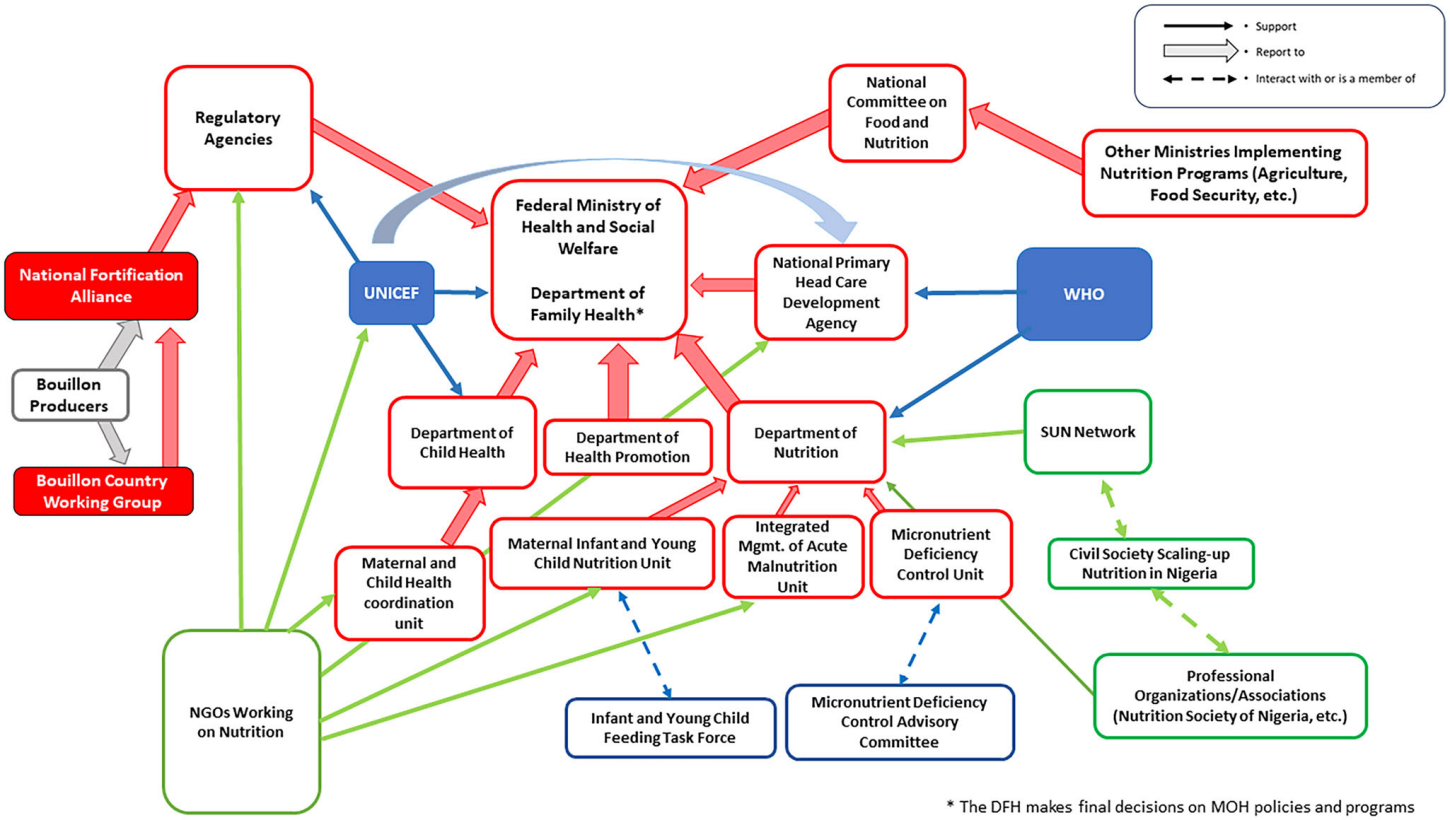
Nutrition policy landscape relevant for bouillon fortification program discussions in Nigeria. Red/red‐outlined figures represent national government organizations/agencies; blue figures represent UN agencies; dark blue figures represent international development agencies; purple figures represent NGOs/international NGOs; green and gray figures represent civil society and industry, respectively; and nutrition groups within technical and financial partner organizations have the potential to interact with all other organizations/agencies identified in the figure. Arrows indicate information and influence pathways. Abbreviations: MOH, Ministry of Health; NGO, nongovernmental organization; SUN, scaling‐up nutrition; UNICEF, United Nations International Children's Emergency Fund; USAID, United States Aid for International Development; WHO, World Health Organization.

In Nigeria (Figure 4), the ultimate authority for setting, enforcing, and funding nutrition policy is the Federal Ministry of Health (FMoH). Tasks associated with each of these general activities were delegated to sub‐FMoH agencies. In the context of fortified foods and condiments, the Standards Organization of Nigeria (SON) was charged with setting fortification standards, while the National Agency for Food and Drug Administration and Control (NAFDAC) and the Federal Competition and Consumer Protection Commission (FCCPC) were responsible for the monitoring and evaluating fortification standards within industry and in the marketplace, respectively, as set by the SON. The NFA was a standing committee commissioned by and reporting to the NAFDAC and was comprised of representatives from national and international industry, academia, NAFDAC, SON, FMoH, nongovernmental organizations (NGOs), and others. The bouillon CWG was commissioned by and reported to the NFA, had a finite lifespan, was comprised of members roughly representative of those that comprised the NFA, and was coorganized by HKI.

**FIGURE 5:**
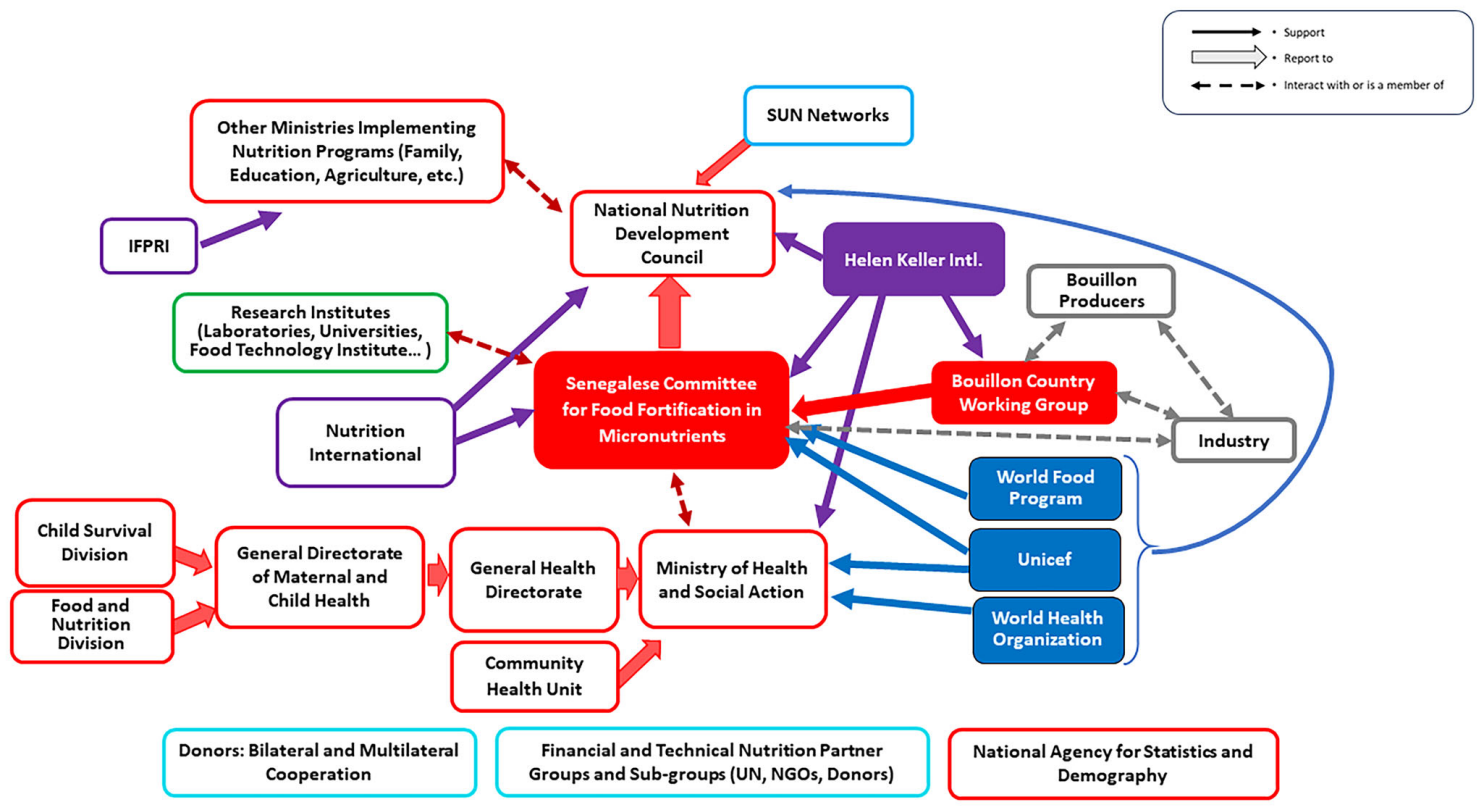
Nutrition policy landscape relevant for bouillon fortification program discussions in Senegal. Red/red‐outlined figures represent national government organizations/agencies; blue figures represent UN agencies; purple figures represent NGOs/international NGOs; green and gray figures represent civil society and industry, respectively; and nutrition groups within technical and financial partner organizations have the potential to interact with all other organizations/agencies identified in the figure. Arrows indicate information and influence pathways. Abbreviations: IFPRI, International Food Policy Research Institute; NGO, nongovernmental organization; SUN, scaling‐up nutrition; UN, United Nations.

In Senegal (Figure 5), the National Nutrition Development Council (CNDN), previously known as CLM, operated under the Prime Minister's jurisdiction and spearheaded the NFA—referred to as COSFAM (Senegalese Committee for Micronutrient Food Fortification). The CNDN's core role involved shaping and executing the country's nutritional policy. Comprised of relevant ministries, local elected representatives, NGOs, and civil society members, this council mirrored the diverse nutrition landscape. Senegalese NFA meetings were infrequent and tied to externally supported projects. Regardless, LSFF programs were part of the Strategic Multisectoral Plan for Nutrition (PSMN), overseen by the CNDN, and were integrated into the monitoring and evaluation framework. Consequently, LSFF programs held greater prominence in Senegal than in Burkina Faso with ongoing performance tracking, although not uniformly followed or supported by all nutrition stakeholder groups. Development partners such as Nutrition International, HKI, GAIN, and the WFP supported NFA initiatives in Senegal. The Senegal bouillon CWG was comprised of a subgroup of COSFAM permanent members and representatives of bouillon importer and producer groups.

The involvement of UNICEF was complex and country‐specific. In the context of Nigeria, the voices of UNICEF and the WHO were somewhat muted vis‐à‐vis the other countries in the study. On the issue of bouillon fortification, national representatives (who were members of the CWG) were initially agnostic (at best) regarding bouillon fortification, mainly because of its sodium content, but eventually tended to agree with the consensus views that emerged within the CWG that: (1) yes, excess sodium intake is a national problem; (2) evidence generated on behalf of the CWG confirmed that for most consumer groups, bouillon was not a large contributor to sodium intake; (3) bouillon is used as an additive to enhance taste and (hence) is unlikely to be used more intensively if fortified; and (4) bouillon should certainly be part of the national strategy for sodium reduction, but that program should include all sources of sodium in the diet.

In the context of Senegal, UNICEF was represented in the CWG and wanted more information on bouillon's contribution to sodium intake prior to supporting the fortification of bouillon. In the end, even when the sodium intake evidence was presented, the UNICEF representatives still chose not to engage in bouillon fortification discussions, citing other new concerns.

In the context of Burkina Faso, UNICEF became engaged only when MINIMOD results related to baseline nutritional inadequacies and the contributions of existing LSFF programs to addressing them were presented at an NFA meeting. Despite interest in these topics, UNICEF refused to engage in bouillon fortification discussions within the CWG.

### Multi‐stakeholder framework^g^

Once the nutrition policy landscapes were identified, with particular focus on entities that were charged with discussing and making decisions regarding multifortified bouillon, specific stakeholders within relevant entities were identified and their access to/interpretation of existing evidence and their evidence needs were assessed. Based on what we discovered, we developed strategies for meeting these needs in timely and impactful ways.

**FIGURE 6:**
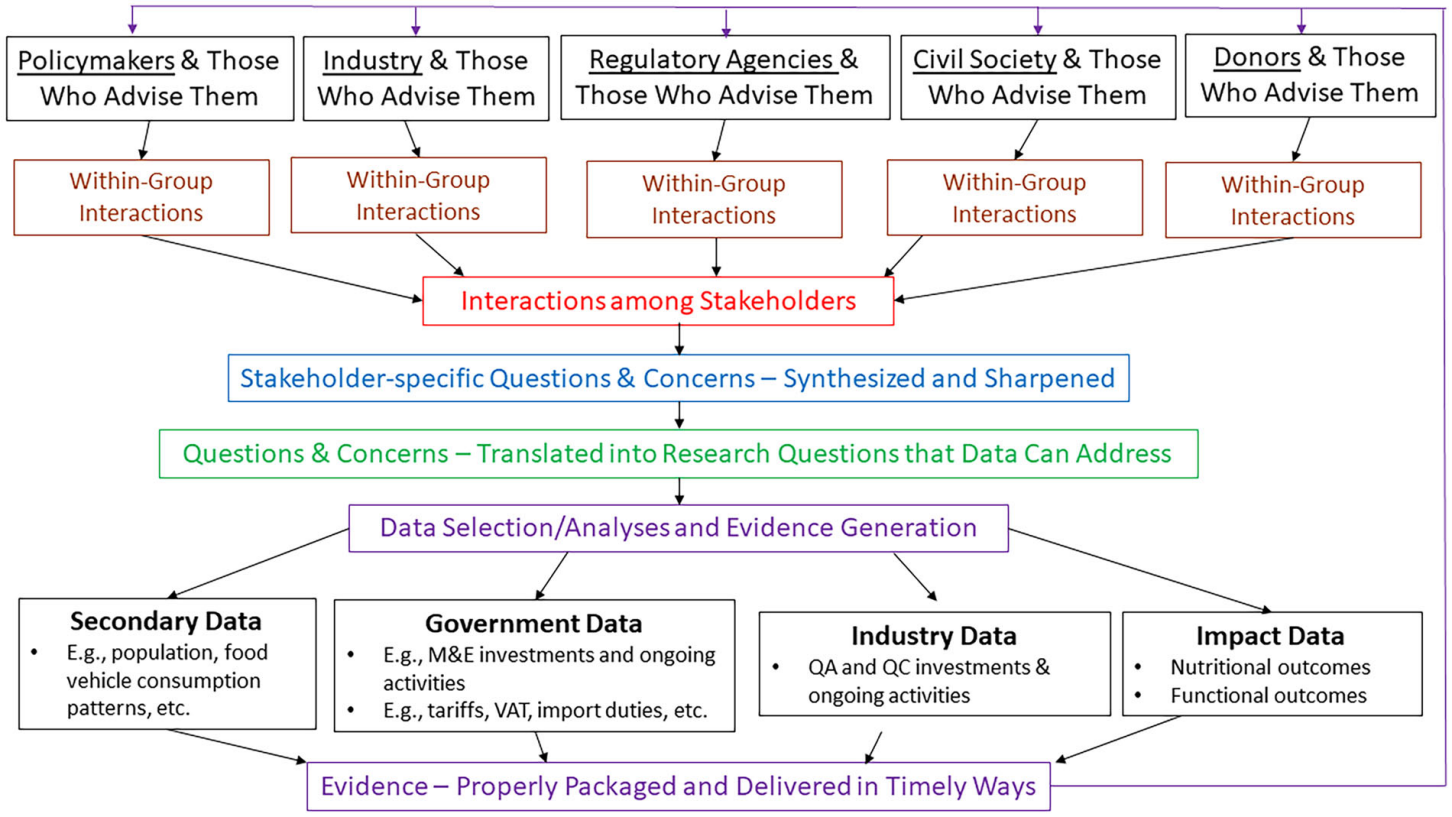
Bouillon fortification program stakeholder groups: from information needs to delivered evidence. QA, quality assessment; QC, quality control; VAT, value‐added tax.

Figure 6 presents a set of generic stakeholders involved in bouillon fortification discussions, highlighting their internal and external information needs. It also illustrates how these needs evolved into research questions, which could be addressed using available data and analytical methods, and how evidence was subsequently delivered to the stakeholders.

This framework was very useful as a tool for probing and cultivating stakeholder‐specific evidence needs; indeed, we learned early in the project that most stakeholder groups had not deeply considered the evidence needed to inform the internal or external discussions that they were involved in, or the data and empirical tools available to produce this evidence. Therefore, the key benefits of this tool were to stimulate discussions around evidence needs, interactions with modeling teams to develop empirical strategies for meeting these needs, and the development of strategies for packaging and delivering elements of the evidence generated to stakeholders. For example, industry representatives were keenly interested in the cost of premix (which they would have to pass onto consumers, with potential consequences for wholesale and retail prices, and for market shares), while public health representatives were keenly interested in nutritional needs and the extents to which alternative combinations and the amounts of fortificants could potentially be included in fortified bouillon and (hence) might help meet nutritional needs.

### New entities and discussion spaces for bouillon fortification

Within, and in some cases alongside, the bouillon CWGs, we established small in‐country teams to assist in model development and to serve as eventual spokespersons for model results and the evidence these provided.

In Burkina Faso, we formed an in‐country MINIMOD core team comprised of HKI and MINIMOD team members. This team engaged with in‐country stakeholders to flesh out Burkina Faso's nutrition policy landscape and to identify additional members for the MINIMOD core team. Expanding the group involved creating terms of reference, presenting the MINIMOD tool to CWG members to garner interest, address queries, and clarify differences among modeling tools that had recently been used in the country. Since models were introduced in the context of exploring bouillon cube fortification issues, initial responses addressed concerns and rumors about this delivery vehicle itself, such as its sodium content and alleged links to health issues, to pave the way for the model presentation and discussions. Initially, the University of California, Davis (UCD) and HKI used the in‐country MINIMOD team to help develop the tool specifically for Burkina Faso, for example, to review and refine selected items from the HCES food list, ensure compatibility with the food composition table, and collect data on LSFF program performance. Throughout 2020, the team held biweekly meetings, transitioning to monthly and eventually to ad hoc meetings in 2021. Despite some familiarity with the tools, stakeholders wanted supplementary information about the food vehicle's contribution to sodium intake before discussing modeling results related to bouillon fortification. Although the team was expanded in 2021 to include representatives from stakeholder groups such as the Nutrition Directorate, UNICEF, WHO, and USAID Advancing Nutrition, attendance declined due to busy schedules, internet issues, and continued concerns regarding sodium intake. To maintain momentum, the smaller HKI‐UCD team persisted with meetings, ensuring model development and connections with the CWG and other groups and institutions through in‐person interactions.

In Nigeria, a subset of CWG members with an interest in and a willingness to contribute to model development was formed. MINIMOD‐Nigeria team meetings were coordinated by HKI and met on a biweekly basis for nearly 2 years, during which key data gaps associated with the nutritional needs/benefits model and the LSFF program cost models were filled, and their preliminary results were vetted. The MINIMOD‐Nigeria team was woven into the CWG formally in 2023, at which time the MINIMOD team began to make formal presentations of the models and of the model results to the CWG, and eventually to the NFA. The team continued to support the CWG and the larger inter‐institutional group that was commissioned to develop the draft Code of Practice (CoP) for voluntary bouillon fortification.^h^

In Senegal, the MINIMOD tool was introduced to nutrition stakeholders in 2019, a year before the bouillon project's launch. Following this, a MINIMOD‐Senegal team was established, complete with terms of reference. Their objective was to secure funding and to create a strategy for developing both a country‐specific tool and the national capacity to operate and update it. This team persisted after the bouillon project began and facilitated its connection with the bouillon CWG. Despite some familiarity with the tools, stakeholders wanted supplementary information about the food vehicle's contribution to sodium intake before discussing MINIMOD results related to bouillon fortification. The MINIMOD‐Senegal team was led by the CLM (later becoming CNDN), which was beneficial for continuity purposes, but was hindered by the busy schedules of its members. Changes in CNDN's affiliation from the Prime Minister's office to the Presidency, along with staff turnovers, caused delays. Meetings in Senegal were infrequent, occurring once per trimester, necessitating presentations for newcomers or refresher presentations due to extended intervals between meetings. However, CNDN members maintained their interest, leading to a more consistent meeting schedule by 2022. The composition of the MINIMOD‐Senegal team changed over time, starting with a larger group and later evolving into a smaller team consisting of CNDN, HKI, and MINIMOD team representatives, ensuring connections with the CWG and other groups and institutions.

### A *lingua franca* established

In all cases, a project‐specific *lingua franca* was needed to facilitate interactions among stakeholder groups, and between stakeholder groups and the research/policy engagement teams. This *lingua franca* was comprised not only of technical terms (e.g., micronutrient vs. fortificant) and concepts (e.g., micronutrient dietary adequacy) that were unfamiliar to many, but also commonly used terms that meant different things to different stakeholders (e.g., optimize). Indeed, quite a lot of time and effort was spent establishing a common vocabulary (in English and in French) that would permit meaningful discussions that could lead to decisions. This common vocabulary was codified and reinforced in the MINIMOD evidence bases and in the PPT slide decks derived from them.

### Country‐specific evidence needs identified and confidence in evidence boosted

**FIGURE 7:**
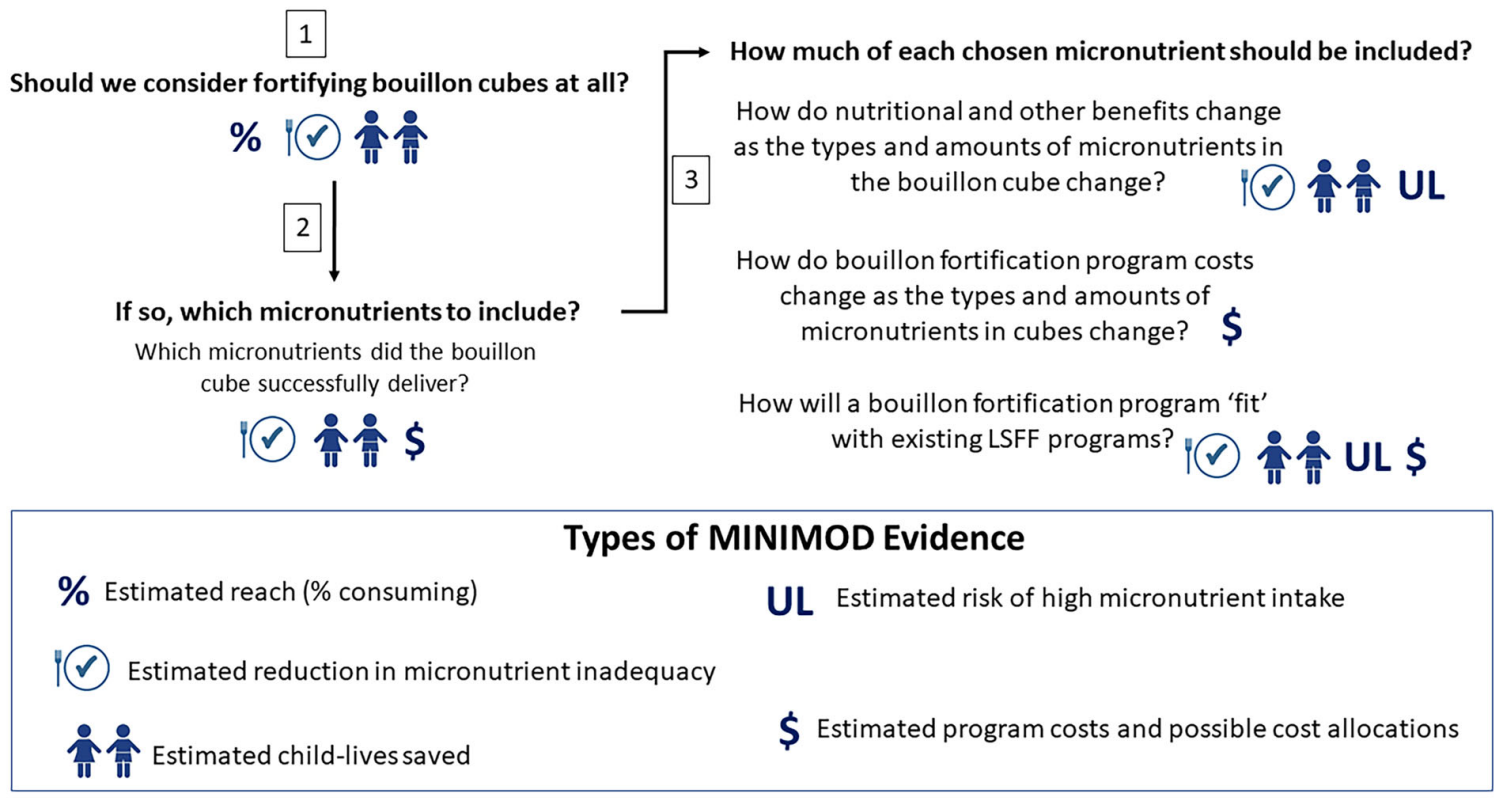
Deciding on the need for and design of a multifortified bouillon program. See Adams et al.,^39^, ^40^, ^41^ Thompson et al.,^42^ and Vosti et al.^43^ for details. Abbreviation: LSFF, large‐scale food fortification.

After establishing in‐country teams and the *lingua franca*, we shifted our focus to developing strategies for identifying the specific evidence needs of stakeholders. When bouillon, a condiment broadly and regularly consumed for decades, was considered for fortification, it triggered several questions about its suitability as a vehicle for micronutrients. Concerns were raised regarding its sodium and monosodium glutamate (MSG) content, and the known links between those ingredients and noncommunicable diseases.^38^ Additionally, some NFA members questioned whether the vitamins added to bouillon would remain intact after food preparation given the cooking and reheating practices in West Africa. While other researchers addressed some of these technical concerns (and their results were delivered using the same discussion spaces that were used to convey modeling results), we focused on fostering understanding of and generating interest and confidence in our modeling research tools, the underlying data, and the evidence they could generate regarding the quantification of nutritional needs and the potential contributions of bouillon fortification to meeting these needs. Figure 7 highlights these steps in the context of a hypothetical multifortified bouillon program.^i^ Step 1 assessed the reach of bouillon compared to other delivery vehicles and the dietary inadequacies in vitamin A, vitamin B12, folate, iron, and zinc. This step required estimates of reach, dietary intake, the contributions of existing LSFF programs (assessed at their current levels of adherence to published standards), and micronutrient requirements (with a particular focus on priority beneficiary groups, e.g., WRA and/or young children). Step 2 was to assess the contributions that a multifortified bouillon cube could make to filling each micronutrient‐specific gap. This step required a series of model simulations under different assumed levels of fortification for each micronutrient, evaluating effective coverage (or the remaining target micronutrient‐inadequate population), child‐lives saved (relevant for vitamin A, zinc, and folic acid), and the proportion of that same population that may be at risk of consuming above the tolerable upper intake level (UL) for micronutrients for which ULs have been established. Step 3 was undertaken alongside the model simulations suggested in Step 2 and focused on estimating the costs associated with alternative combinations and levels of fortificants in the premix; the start‐up, operational, and M&E costs associated with a bouillon fortification program; and the potential allocation of costs across stakeholder groups.

### Models and supporting data introduced

Models were mysteries to many stakeholders, who were generally unaware of the data that supported the models and were very unaware of the core assumptions that underpinned the models, and hence were initially highly skeptical of model results. Moreover, all stakeholders had their own (and sometimes conflicting) views regarding the issues at hand, and in some cases, their own data and observations to support their views. Therefore, the first step was to discover what these stakeholder‐specific views were regarding the issues at hand (e.g., micronutrient inadequacies) and what supported these views. This required long discussions during which stakeholder views were more clearly defined, thereby identifying what was known (and how) and what evidence gaps remained. The next step was to systematically introduce the models and their supporting data and assumptions to all stakeholders, initially relying on models developed for other country contexts but still sufficient to make key general points, while routinely circling back to stakeholders’ views to coax them into mindsets that would permit the strategic introduction of new data, methods, and evidence in the contexts of their own countries.

To introduce the models and their potential for producing evidence to inform decision‐making in the context of bouillon fortification, comprehensive presentations were conducted in all three countries. These presentations encompassed a range of critical aspects including the fundamental concepts underpinning each model, the sources of data to be utilized, the key assumptions included in each model, and the types of results each model could generate. We started by presenting the models and modeling results for Cameroon, and gradually introduced data and results pertaining to project countries as they became available.

Several core objectives were achieved during the introduction of MINIMOD models. First, we discovered more about the objectives and priorities of each stakeholder group, for example, some stakeholders were very concerned about specific subgroups of national populations (e.g., WRA and young children, or individuals residing in poor households), while others were concerned about populations in particular geographic areas within countries. Second, we introduced alternative definitions and metrics of programmatic success, some of which were new to some stakeholders. For example, terms such as reach, coverage, effective coverage, and cost‐effectiveness were introduced and discussed, and the groundwork was established for using The Lives Saved Tool (LiST) to translate effective coverage into child‐lives saved. Third, and perhaps most important, the modeling team became aware of the modifications that needed to be made to the models to meet stakeholders’ evidence needs and began to develop strategies for packaging modeled evidence to make it absorbable by stakeholders. For example, it became very clear that stakeholders needed subnational spatial depictions of micronutrient needs and the impacts of alternative bouillon fortification programs on those needs. The MINIMOD team responded by producing maps that depicted this evidence. Fourth, in order to enhance confidence in and ownership of the model results that would eventually emerge, we were careful from the outset to identify the models’ limitations. For example, while changes in dietary habits were acknowledged in all countries, we made clear that the diets underpinning the MINIMOD needs/benefits model were frozen as of the date of the HCES survey data collection—diets varied substantially across households, which reassured stakeholders who were concerned about the nonuniform spatial patterns of the potential benefits of bouillon fortification, but diets for any *given* household did not change over the model's 10‐year simulation period. Or, although fortificant prices are known to vary temporally, fortificant prices contained in the MINIMOD LSFF program cost model were constant over the model's 10‐year simulation period. To address these limitations, it was important to share the results of sensitivity analyses that demonstrated that the core modeling results and their policy implications were robust to uncertainty in these and other parameters that could influence the estimated costs and benefits of alternative LSFF program options.

### Co‐developed models

Once the MINIMOD models had been introduced and the reasons for developing and using them were understood, we began the process of co‐developing the models for each country. Small teams comprised of individuals interested in and available to participate in model development were formed in each country, with some cross‐team linkages. In all cases, the research teams used existing, recent HCES data to address issues related to micronutrient inadequacies and constructed LSFF program cost models to address efficiency and affordability issues (for model details, see Adams et al.,^39^, ^40^, ^41^ Thompson et al.,^42^ and Vosti et al.^43^). The country teams, in close partnership with other stakeholders such as national statistical institutes, played pivotal roles in refining the definitions of certain food items found in the HCES questionnaire food lists, thereby assisting in the mapping of HCES food items into food composition tables. National teams also facilitated the translation of nonstandard measurement units into internationally recognized units to better quantify household food intakes. National teams contributed to the refinement of assumptions related to existing LSFF program performance, actively seeking locally available program performance data or relying on information from the Global Food Database (GFDx) platform. Teams were also key in identifying the expected levels of performance of the hypothetical bouillon fortification programs. For example, given the porous nature of international borders in Nigeria, the in‐country team did not believe that regulatory agencies could manage to secure more than 75% compliance with whatever standards were generated; this upper bound on compliance for bouillon fortification was integrated into all MINIMOD‐SD and cost model simulations.

Once these issues were addressed, initial sets of own‐country–specific results were generated and shared with in‐country collaborators for review and comments.

During the model development process, the demand for different types of evidence became more concrete in country‐ and stakeholder‐specific ways. For example, in Senegal, where rice consumption is common and where the multifortification of rice is being considered, additional model components had to be developed to demonstrate the potential contributions of different levels of bouillon fortification in the presence of a hypothetical rice fortification program. In all countries, concerns regarding rural versus urban populations in regard to both micronutrient inadequacies and program impacts prompted the addition of model components that could generate the needed evidence.

One of the key takeaways from our model co‐development experience was the importance of repetition. We found it necessary to revisit and reiterate many of our underlying assumptions and the process by which results were generated. This repetition was prompted by several factors. First, the online presentation format posed a challenge as it required stakeholders to absorb a substantial amount of new information during virtual presentations. Additionally, the composition of in‐country teams changed over time, requiring revisiting key points. Furthermore, the attention of stakeholders was not consistently at its peak during the discussions partly due to challenges related to bouillon as a vehicle, which generated ongoing concerns and distractions. Creating French‐language presentations for Burkina Faso and Senegal was crucial for maintaining engagement and enhancing comprehension. This step may seem straightforward, but it is worth noting that many available resources and evidence were not in all major languages.

### Required evidence generated and delivered

Once identified, the elements of Figure 7 provided the focus and the structure of the evidence that was produced for each country. More specifically, for each country, the MINIMOD evidence bases were comprised of: (1) initial sets of assumptions regarding the standards and performances of existing LSFF programs, hypothetical improved LSFF programs, and hypothetical bouillon fortification programs; (2) assessments of the geographical and socioeconomic reach of alternative delivery vehicles; (3) estimates of micronutrient adequacy for young children and for WRA for five micronutrients ignoring existing LSFF programs; (4) the contributions of existing LSFF programs to reducing micronutrient inadequacy; (5) the additional contributions of bouillon fortified at 15% and 30% of Codex Nutrient Reference Values (for WRA consuming 2.5 g/day); (6) risks of consuming above the ULs for relevant micronutrients; (7) child‐lives saved by bouillon fortification at various levels and combinations of zinc, vitamin A, and folic acid; and (8) bouillon fortification program cost, cost‐efficiency, and cost‐effectiveness. For examples of the specific types of evidence generated by the MINIMOD modeling team, see Adams et al.,^39^, ^40^, ^41^ Thompson et al.,^42^ and Vosti et al.^43^

The country‐specific MINIMOD evidence bases were presented in structured and indexed PPT slide decks that contained over 100 slides each. Evidence on micronutrient inadequacy and the contributions of programs to address it (individually and in combinations) was provided for the national and subnational levels; evidence on the impacts of LSFF programs on child‐lives saved was provided at the national level. The volume of evidence was too large for members of the CWGs to effectively process, so the MINIMOD teams worked with CWG participants to identify and repackage key elements of the overall evidence bases that were deemed to be most useful for bouillon fortification policy discussions. These more focused sets of evidence were delivered in smaller PPT slide decks (in English or French, as appropriate) and in the form of Research Briefs.^j^

In Nigeria, it was determined by the CWG that vitamin A would not be considered for bouillon multifortification, so new model simulations were run, and new evidence was provided to identify the consequences of this decision in terms of nutritional and mortality benefits, and program costs. In Burkina Faso, the CWG requested evidence on alternative food vehicles (rice and maize flour) due to persisting resistance to the use of bouillon cubes as a micronutrient delivery vehicle by some stakeholders. Comparing the reach of fortifiable rice and maize flour to that of bouillon demonstrated the wisdom of continuing to consider bouillon.

It is important to note that a productive symbiosis emerged. Policy engagement activities were fundamental to the development of analytical tools that could respond to specific stakeholders’ evidence needs. Tool development also influenced the nature and content of policy discussions. For example, stakeholders struggled with the tabular presentation of model results, so researchers recast results in the form of maps highlighting the national and subnational results of, say, alternative bouillon fortification programs. To take another example, this time in the opposite direction of information flows and influence, modelers struggled with the incomplete sources and amounts of specific micronutrients in diets (especially vitamin A), so stakeholders provided guidance on these and other key model parameters. In the end, policy engagement activities helped generate tailored and timely evidence to inform discussions of bouillon fortification programs.

### Impacts of new evidence on bouillon fortification policy discussions

Several key outcomes emerged from evidence generated and delivered to stakeholders. Recall that the modeled evidence generated/delivered in the context of this project always was set alongside other types of evidence, for example, technical information regarding the impacts of alternative fortification options on organoleptic characteristics of cubes and foods cooked using cubes, some of which was not publicly available (e.g., industry costs).

In all countries at the outset of the bouillon project, discussions within the CWGs were hampered by a lack of recent evidence on micronutrient needs based on diets without considering existing fortification programs, the contributions of existing LSFF programs to meeting nutritional needs, the additional potential contributions of hypothetical bouillon fortification programs to meeting nutritional needs, and for some micronutrients, the reduction in child mortality. Evidence on the costs and cost‐effectiveness of alternatives to hypothetical bouillon fortification programs, including which stakeholder groups would bear these costs, was also missing, as were estimates of the contributions of bouillon to total sodium intake. The evidence provided for each country helped fill the nutritional and cost evidence gaps, and studies were commissioned to address the sodium issue.

Policy engagement activities were structured and timed to deliver key pieces of evidence as they became available to CWGs, and in doing so, made discussions more concrete and productive, although not necessarily less noisy.

There was general recognition of the potential of fortified bouillon (on its own and relative to existing LSFF programs) to reduce micronutrient inadequacies broadly and equitably. For selected micronutrients (e.g., vitamin A, zinc, and folic acid), the potential to save children's lives was also demonstrated. Although the inadequacy‐reducing and child‐life–saving benefits varied across countries due to underlying diets and to bouillon consumption patterns, there was a deeper understanding of why bouillon could be a promising candidate as a vehicle for delivering needed micronutrients.

It became clear that bouillon was more impactful and cost‐effective at delivering some micronutrients than others. For example, due to absorption and fortificant cost issues, iron was much less cost‐effective than, for example, vitamin B9.^k^ This provided opportunities for discussions regarding the formulation of premixes for bouillon and for other food vehicles, too. To date, discussions regarding the premixes used in existing LSFF programs have been somewhat muted by international organizations.

Subnational sodium studies in all countries confirmed the relatively small contributions of bouillon to overall sodium intake: Burkina Faso (∼15%),^44^, ^45^ Nigeria (6.9% [males in Ogun] to 40.8% [females in the Federal Capital Territory]),^36^ and Senegal (∼15%).^46^, ^47^ These contributions are low relative to nonbouillon sources of salt in foods prepared at home, prepared foods (e.g., breads), and foods consumed outside the home. This evidence helped reduce the attention paid to this issue in the context of bouillon fortification discussions, and strengthened calls for broad, national discussions of policies to reduce sodium intake, which should also include bouillon.

In Nigeria, elements of the evidence were actively used to support the development of a CoP for multifortified bouillon, demonstrating the practical application of our findings in this context. More specifically, the NFA requested that the CWG prepare a CoP for the fortification of bouillon. The CoP, which provided guidance to industry partners that choose to voluntarily fortify bouillon products, would serve as a first step toward an eventual mandatory bouillon fortification standard. A consultant and one assistant were hired to prepare a draft CoP (the MINIMOD team helped draft the Terms of Reference for the consultant), which put a heavy emphasis on the need to specifically identify the types and amounts of fortificants that would be included in a multifortified bouillon cube. The MINIMOD team had many technical discussions with the consultant, providing suggestions regarding the micronutrient content to be included in the draft document. The CoP requiring approximately 15% of Codex nutrient reference values for iron, zinc, B12, and folic acid was recently formally adopted by the FMoH.^l^

### Co‐benefits of evidence generation and policy engagement activities

In addition to the direct benefits of bouillon fortification policy discussions, model development, evidence generation, and policy engagement activities also generated other indirect benefits.

In all countries, model results (which identified the low performance of some existing LSFF programs) highlighted the need to reinforce the monitoring of LSFF programs to ensure that existing fortification programs and eventually bouillon programs would deliver the benefits that they were designed to generate. As a result, in Burkina Faso, for example, the NFA put in place a task force mandated to develop a national plan for monitoring and evaluating LSFF programs. USAID Advancing Nutrition supported the process, and the finalized plan was awaiting adoption by the government.

In all countries, it was recognized that the capacity of LSFF national stakeholders charged with overall program coordination and monitoring needed to be expanded and strengthened. Related, it became clear that in Senegal and Burkina Faso, there were no regular LSFF program reviews scheduled; instead, reviews are mainly carried out in the context of projects proposed by development partners.

In all countries, discussions regarding the ownership of research tools and evidence arose. Questions arose regarding the practical definitions of ownership (of what, by whom), from the ownership and use of the existing evidence bases and the results they contained, to the ownership and continual use of the MINIMOD tools themselves. Senegal, in particular, was keen to move forward with the investments (in human capital, mainly) required to secure ownership of, and to make use of, the evidence and the tools. In Nigeria, a first step in tool ownership focused on the LSFF cost models; the NAFDAC was considering becoming the institutional home for that set of models.

All countries also saw the opportunity to integrate the types of nutrition analysis being done to support bouillon fortification discussions into the periodic analyses undertaken by national statistical institutes on HCES data to support the monitoring and planning of micronutrient intervention programs, and the development of new programs.

In Senegal and Nigeria, the evidence prompted discussions regarding the possibility of discontinuing vitamin A supplementation programs in some areas of the country if LSFF programs were to perform as intended.

In all countries, discussions shifted from a focus on particular micronutrient delivery vehicles or platforms to a more integrated focus on sets of micronutrient intervention programs (LSFF and supplementation programs), and to the consideration of the nutritional and other benefits and the costs of program packages in their discussions. Relatedly, modeled evidence helped operationalize the concept of multisectoral planning, which was trending in the lexicon but not well understood. This emerging, integrated focus faces challenges, in part because development partners often support specific nutrition intervention programs rather than sets of them.

## DISCUSSION

Evidence disconnected from policy‐making processes will not likely bring about desired changes in policies. There are several root causes of these disconnects.^48^ Analysts (i.e., research groups, national statistical institutes, UN/NGOs data analysts, and others producing evidence for decision‐makers) may not know the types of evidence that stakeholders need to make informed decisions regarding the choices of and the design of LSFF programs. In addition, in‐country analysts (in particular) may not have the latitude and the resources to generate needed evidence. Finally, the mechanism for introducing evidence into policy discussions may not function well, or even exist. Moreover, it is hard to know *a priori* the amounts or types of stakeholder‐specific evidence that will trigger the hoped‐for buy‐in or expected policy response; in practice, this may only become apparent as policy discussions evolve.

Many groups have wrestled with these issues in the context of nutrition policy discussions,^13^, ^14^, ^16^ and their efforts helped provide an overall framework for our work, which extends and enriches previous studies. This paper also contributes to the emerging literature on the co‐development of food, food environment, nutrition, and health programs and policies, touching on each of the co‐development opportunities identified in that literature—namely, co‐deciding on what to pursue and why, co‐planning, co‐designing intervention programs, co‐evaluating programs, co‐disseminating evidence and information, and the co‐implementation of programs and policies.^49^, ^50^, ^51^, ^52^, ^53^, ^54^ More specifically, the paper describes a strategy, implemented in Burkina Faso, Nigeria, and Senegal, for policy engagement alongside evidence generation to inform discussions around the multifortification of bouillon. The paper also reports on the benefits and co‐benefits of the policy engagement and evidence generation activities, and on the challenges faced.

The nutrition policy issues we faced and the general approach that we adopted were not new. Indeed, learned societies (e.g., the Society for Implementation Research in Nutrition) have been developed to address these issues. That said, this paper breaks ground on new policy engagement and research in several ways. First, we focused narrowly on a novel vehicle for delivering micronutrients, bouillon, and new product and input market settings, policies, and the controversies that came with it. Second, we paid particular attention to identifying and engaging with specific stakeholder groups to discover their concerns and evidence needs (as they related to bouillon in particular, but also in the broader context of LSFF programs), and to co‐develop strategies for addressing both. Third, we tailored research to generate the evidence needed by all stakeholder groups, even when stakeholder‐specific needs did not overlap. Fourth, we focused on final outcomes (e.g., modeled reductions in micronutrient inadequacies and child‐lives saved) rather than on intermediate outcomes (e.g., LSFF program reach). Fifth, we delivered evidence on estimated intervention program costs. Sixth, if arenas for discussion/debate/dissemination did not exist, we created them. Seventh, we persisted and reiterated—ours was a multiyear research and policy engagement project during which we maintained continual contact (virtual and in‐person) with collaborators and stakeholders, despite expected lulls in interest and action by some stakeholder groups and the formidable challenges posed by the COVID‐19 pandemic. Finally, and as a result of the second through seventh factors just noted, we created trust. Midway through the project, it became clear that the evidence transferred to stakeholders and used by them bore their stamps of approval.

In all of the countries, the initial obstacle to discussions around bouillon fortification was the negative perceptions of the food vehicle itself. These perceptions slowed the progress of discussions and undermined confidence in MINIMOD evidence; to some stakeholders, we were seen initially as bouillon promoters. Studies were commissioned in each country to discover bouillon's contributions to total sodium intake. These studies, which generally reported that bouillon contributed to ∼15% of total sodium intake, served to quiet some critics of bouillon as a delivery vehicle and to elevate discussions of sodium intake reductions to a national, multi‐vehicle level. Concerns related to MSG and other rumored negative effects of bouillon also slowed and complicated bouillon fortification discussions. In addition, in all countries, LSFF programs as a strategy for addressing micronutrient deficiencies often remain overlooked within the broader nutrition community where the current emphasis tended to be on micronutrient supplementation, dietary diversification, or point‐of‐use fortification.^48^ Despite these challenges, discussions and decisions around bouillon fortification did proceed, although progress was not uniform across the three project countries.

Progress was most significant in Nigeria, where experience with LSFF programs was relatively longer and broader, and where a strong and active NFA formed and managed a determined bouillon CWG that valued evidence, commissioned studies to fill important evidence gaps, and used evidence to guide their deliberations. The presence of former or current academics and scientists on the CWG greatly influenced the types and tones of their deliberations; the presence of government and industry representatives facilitated the identification of strategies for developing and implementing a voluntary CoP for bouillon fortification and a pathway toward mandatory fortification. Perhaps most fundamentally, there was a shared and clearly articulated commitment by the MoH to addressing micronutrient inadequacies.

Progress was limited in the case of Burkina Faso for an array of reasons. Bouillon CWG participants came from various backgrounds and some with limited understanding of LSFF programs in general. This point of departure slowed discussions because basic information needed to be provided. CWG member turnover also hampered progress, requiring that concepts, data, tools, and even project objectives be reintroduced and redebated on several occasions. Seeking consensus regarding discussions around bouillon fortification was also hampered by the insistence by some CWG members that issues related to sodium intake had to be discussed and resolved prior to moving forward. Overall, the commitment within the MoH to addressing micronutrient inadequacies was less central. That said, national bouillon manufacturing and safety standards, aligned with those of neighboring countries, were adopted in 2021. The discussion resulted in a consensus that current industry‐led voluntary bouillon fortification should be regulated. The Agence Burkinabé de Normalisation (ABNORM) and the Ministry of Trade remain prepared to integrate regional fortification guidelines into existing national frameworks once these guidelines are developed, but no national plan for bouillon fortification has yet been proposed.

In Senegal, members who would eventually comprise the bouillon CWG were exposed to the MINIMOD tool and evidence before the bouillon project began, so in some ways, the Senegalese had a head start on understanding and using the evidence generated by the MINIMOD models. However, discussions routinely circled back to the desire for building national capacity to use the models (which the bouillon project was not designed to support), thereby slowing efforts to generate and use the national MINIMOD evidence base. In Senegal, the commitment to addressing micronutrient inadequacies was significant, but institutional and other issues made getting traction on bouillon fortification discussions challenging. However, the CWG and the Standards Bureau now support fortification aligned with the Economic Community of West African States (ECOWAS) regional bouillon standard (when it emerges). In addition, with backing from the MoH, discussions are underway to adopt reduced‐sodium fortified bouillon standards that incorporate acceptable levels of vitamin A, zinc, and folic acid; these acceptable levels have not yet been determined. Local manufacturers have shown growing interest, especially following advocacy efforts highlighting how fortification aligns with national nutrition goals.

In both Burkina Faso and Senegal, the National Food Fortification Alliances demonstrated limited organizational structure, characterized by an absence of clear roles and responsibilities and irregular meeting schedules. This institutional fragility created an environment that was less conducive to fostering the demand for and acceptance of MINIMOD evidence. Investments could be made to strengthen these organizations, but substantial political commitments would be required to do so sustainably.

Ownership was an issue that was introduced at the outset of the project in all countries and routinely discussed. Ideally, every country wanted to be self‐sufficient in the use of the models, the periodic generation of updated evidence, and the as‐needed distillation of elements of the evidence bases to support policy discussions around LSFF programs generally. We fully supported that aspiration and led discussions regarding the meaning of ownership (ownership of what research processes, research results, research tools, etc.) and of the types of investments that would need to be made (mainly in human capital) to achieve various degrees of ownership. We found that achieving clarity on these issues greatly facilitated progress on model development and the generation and use of evidence, and also helped countries chart courses for achieving desired levels of ownership.

A set of best practices regarding policy engagement and research emerged from this three‐country project; these follow the general literature on these issues, but provide more details. First, it is important to identify and engage with specific stakeholder groups to discover their concerns and evidence needs, and to codevelop strategies for addressing both. Second, researchers and policy engagement specialists should work together to generate the evidence needed by all stakeholder groups, even when stakeholder‐specific needs do not overlap. Third, there are huge benefits to focusing on measures of impact that resonate with all stakeholders; our partial focus on child‐lives saved was particularly useful in securing and sustaining the interest of most stakeholder groups, especially beyond nutritionists. Fourth, if arenas for discussion/debate/dissemination do not exist, create them while also understanding that time has value, so the benefit of new committees, meetings, and so on must be clear and compelling. Fifth, while it is very important to be specific about what is meant by *take policy action*, in the end, the MOH will have to sign legislation and technical documents to create and fund a bouillon fortification program. Many other actions must be identified and taken beforehand, and all of these actions require planning and the delivery of evidence. Sixth, as always, it is important to establish short‐, medium‐, and long‐term project objectives and adhere to them. Progress on projects as complicated as bouillon fortification will take time and demonstrating the commitment of researchers and policy engagement teams to stay the course, and to have that course actually generate incremental progress, truly are necessary conditions for success. Seventh, it is critical to develop and continually engage with trusted in‐country allies. These allies are individuals or sets of them who are informed of policy processes value evidence, have a shared commitment to the ultimate project objective (in this case, reducing micronutrient inadequacies), and can commit to dedicating the time required to effectively collaborate.

Countries do not exist in commercial, political, or institutional vacuums. All of these factors influenced the nature and the speed of discussions regarding bouillon fortification. From a commercial perspective, no bouillon was being produced in Burkina Faso and much of the bouillon consumed in Senegal was produced elsewhere; the opposite was true for Nigeria. Therefore, Burkina Faso and Senegal may be in the position of policy‐takers rather than policymakers in the context of bouillon fortification standards, with national policy actions focusing on monitoring bouillon imports rather than bouillon production facilities. On the political front, regional bodies exist (e.g., West African Health Organization, West African Economic and Monetary Union, and ECOWAS) that are relevant for supra‐national discussions, especially when fortified products are traded internationally. Institutionally, NGOs and international NGOs hold great sway in determining which food/condiment vehicles are chosen and what premix formulas are used. The vehicles and premixes proposed by these groups tend not to consider national contexts, thereby sometimes introducing friction and delays into national discussions around these choices.

Finally, it is worth noting that there are programs that have seamlessly integrated problem assessment, program design and implementation, and impact assessment, for example, NTD^55^ and the Expanded Programme on Immunization.^56^ These programs benefited from clear objectives, careful planning and program integration, and consistent funding at required levels. They also had global leads that brought all global partners around the table to establish common guidelines and organize support to countries in coherent ways. Current increases in funding to support LSFF programs lack such organization and focus, thereby leading to gaps, competition, and duplication. More coordination and collaboration are needed in the context of LSFF programs; NTD and similarly structured and managed programs can provide examples of how these objectives can be achieved.

The activities and results reported here have several limitations. First, the study focused mainly on the hypothetical case of multifortified bouillon. Ongoing work will confirm the biological efficacy of an experimental multifortified bouillon cube containing five micronutrients in the context of northern Ghana.^57^ Second, while experimental multifortified bouillon cubes have been produced and found acceptable,^58^ commercial versions of multifortified bouillon cubes remain to be produced, and technical and commercial challenges will be faced and must be overcome.^59^ Third, some of the study's great strengths may pose limitations to others seeking to replicate the activities reported here. Namely, policy engagement specialists enjoyed privileged access to and close collaboration with the modeling team that was able to generate new, tailored evidence to meet stakeholders’ needs; this required substantial financial support and time commitments that other groups might not have. Fourth, and related, the policy engagement activities were nested within a larger, international project that provided continuity and institutional support that was essential to our success. Not all policy engagement teams will be so fortunate. Fifth, although the policy engagement team had to create and manage new discussion forums, they benefited to different degrees from existing national organizations and were, therefore, able to insert their messages and evidence into these organizations that had the mandates and the political clout to move the bouillon fortification agenda forward. The absence of such organizations would pose additional challenges.

## CONCLUSION

Beginning essentially from scratch, it was possible in the period of a few years to identify a promising micronutrient delivery vehicle; identify evidence gaps; develop the evidence that met the needs of all stakeholder groups; deliver key elements of that evidence base to stakeholders in ways and at times that promoted the delivery vehicle and contributed it to concrete fortification program design; secured the buy‐in from stakeholders; and ultimately turned an idea into concrete policy action. This was precisely what played out in the context of bouillon fortification in Nigeria.^52^ This success can be attributed to a shared commitment to a common objective, close collaboration between policy engagement specialists and researchers, the establishment of discussion spaces for bouillon that were linked to existing organizations that managed LSFF programs, effective participation and collaboration by all stakeholders in policy discussions, establishing and implementing a strategy that had voluntary fortification standards for bouillon as its medium‐term objective, and having sufficient resources to get these jobs done. Replicating this success in Burkina Faso and Senegal will require overcoming challenges and ensuring that the key ingredients to policy success are in place; this is achievable in both contexts with sufficient time, resources, and political will.

## AUTHOR CONTRIBUTIONS

A.T., J.W.S., and S.A.V. designed the study and developed the methods. A.T., J.W.S., M.B., F.I., K.K., and A.O. managed in‐country and regional policy engagement activities. A.T., J.W.S., and S.A.V. prepared the first and subsequent drafts of the paper. All authors contributed to revisions of the manuscript, and read and approved the final manuscript.

## COMPETING INTERESTS

All authors have no competing interests to declare.

## PEER REVIEW

The peer review history for this article is available at https://publons.com/publon/10.1111/nyas.70006.

## Data Availability

The data that support the findings of this study are available on request from the corresponding author. The data are not publicly available due to privacy or ethical restrictions.

## Footnotes

a Some aspects of the literature on scaling‐up programs and policies outside of the health sector are also relevant.^60^, ^61^

b For project details, see https://globalnutrition.ucdavis.edu/research/research_proj ^62^ and https://minimod.ucdavis.edu/.^63^

c The terms *evidence* and *evidence needs* should be interpreted broadly, the former to include all information, privately held or publicly available, that could be relevant for policy discussions related to the multifortification of bouillon, and the latter to include evidence needs that became apparent during policy discussions.

d GFDx website (https://fortificationdata.org/), accessed February 15, 2024.

e Evidence needs were sometimes unknown to specific stakeholder groups, for example, policymakers were generally unaware of the costs of imported premixes, and industry was generally unaware of the nutritional and child mortality benefits associated with fortification.

f An example of the semi‐structured survey instrument is available upon request.

g The importance of and methods for identifying multiple stakeholders has been noted by many others, and tools have been developed to assess their relative importance in resource use and decision‐making (e.g., https://netmap.wordpress.com/). Our contributions are the focus on bouillon fortification programs, the identification of evidence needs of stakeholders in this context, and the distillation of these needs into research questions that could be addressed with available data.

h The Code of Practice, which included ∼15% of Codex nutrient reference values in 2.5 g of product for iron, zinc, vitamin B12, and folic acid, was officially adopted by the Nigerian Government on September 17, 2024. https://www.youtube.com/watch?v=i4CZn9V4INE

i For guidance on methods and modeled results associated with each step in Figure 7, see Adams et al.,^39^, ^40^, ^41^ Thompson et al.,^42^ and Vosti et al.^43^

j Research Briefs are available at https://minimod.ucdavis.edu/publications/.

k The bouillon consortium chose micronized FePP as the iron fortificant, which was included in all MINIMOD model simulations. Additional simulations focusing on electrolytic iron suggest that fortificant was less expensive and might be more absorbable than FePP, making electrolytic iron more cost‐efficient and cost‐effective than FePP. That said, technical and other challenges might need to be faced, and these could vary by the amounts of either fortificant included in premixes.

l Note that vitamin A, which modeled results demonstrated would be impactful and cost‐effective in reducing child mortality and carries other benefits, was not included in the CoP. Reasons provided by the CWG for excluding vitamin A were that Nigeria already had five LSFF programs delivering this micronutrient, and there were issues around vitamin A losses during product storage and cooking that needed to be addressed.

## References

[1] Passarelli, S. , Free, C. M. , Shepon, A. , Beal, T. , Batis, C. , & Golden, C. D. (2024). Global estimation of dietary micronutrient inadequacies: A modelling analysis. Lancet Global Health, 12(10), e1590–e1599. 39218000 10.1016/S2214-109X(24)00276-6 PMC11426101

[2] Han, X. , Ding, S. , Lu, J. , & Li, Y. (2022). Global, regional, and national burdens of common micronutrient deficiencies from 1990 to 2019: A secondary trend analysis based on the Global Burden of Disease 2019 study. E Clinical Medicine, 44, 101299. 10.1016/j.eclinm.2022.101299 PMC8850322 35198923

[3] Stevens, G. A. , Beal, T. , Mbuya, M. N. N. , Luo, H. , & Neufeld, L. M. (2022). Micronutrient deficiencies among preschool‐aged children and women of reproductive age worldwide: A pooled analysis of individual‐level data from population‐representative surveys. Lancet Global Health, 10(11), e1590–e1599. 10.1016/S2214-109X(22)00367-9 36240826 PMC10918648

[4] Food Fortification Initiative, Future Fortified, and GAIN . (2024). Large‐scale food fortification is a safe and cost‐effective strategy to improve nutrition. https://www.gainhealth.org/sites/default/files/publications/food‐fortification‐brief‐final‐6‐21‐24.pdf

[5] 76 World Health Assembly . (2023). Seventy‐sixth World Health Assembly: Geneva, 21–30 May 2023: resolutions and decisions, annexes. Geneva: World Health Organization. https://iris.who.int/handle/10665/376760

[6] USAID . (2024). Large‐scale food fortification building nutritious, resilient & sustainable food systems. https://www.usaid.gov/sites/default/files/2022‐05/USAID_LSFF_FS_V5_508.pdf

[7] WHO . (2024). Food fortification recommendations. https://www.who.int/health‐topics/food‐fortification#tab=tab_2

[8] Olson, R. , Gavin‐Smith, B. , Ferraboschi, C. , & Kraemer, K. (2021). Food fortification: The advantages, disadvantages and lessons from sight and life programs. Nutrients, 13(4), 1118. 10.3390/nu13041118 33805305 PMC8066912

[9] Mkambula, P. , Mbuya, M. N. N. , Rowe, L. A. , Sablah, M. , Friesen, V. M. , Chadha, M. , Osei, A. K. , Ringholz, C. , Vasta, F. C. , & Gorstein, J. (2020). The unfinished agenda for food fortification in low‐ and middle‐income countries: Quantifying progress, gaps and potential opportunities. Nutrients, 12(2), 354. 10.3390/nu12020354 32013129 PMC7071326

[10] Neufeld, L. M. , Baker, S. , Garrett, G. S. , & Haddad, L. (2017). Coverage and utilization in food fortification programs: Critical and neglected areas of evaluation. Journal of Nutrition, 147(5), 1015S–1019S. 10.3945/jn.116.246157 28404835 PMC5404214

[11] Aaron, G. J. , Friesen, V. M. , Jungjohann, S. , Garrett, G. S. , Neufeld, L. M. , & Myatt, M. (2017). Coverage of large‐scale food fortification of edible oil, wheat flour, and maize flour varies greatly by vehicle and country but is consistently lower among the most vulnerable: Results from coverage surveys in 8 countries. Journal of Nutrition, 147(5), 984S–994S. 10.3945/jn.116.245753 28404836 PMC5404213

[12] Bauer, M. S. , Damschroder, L. , Hagedorn, H. , Smith, J. , & Kilbourne, A. M. (2015). An introduction to implementation science for the non‐specialist. BMC Psychiatry, 3(1), 32. 10.1186/s40359-015-0089-9 PMC4573926 26376626

[13] Theobald, S. , Brandes, N. , Gyapong, M. , El‐Saharty, S. , Proctor, E. , Diaz, T. , Wanji, S. , Elloker, S. , Raven, J. , Elsey, H. , Bharal, S. , Pelletier, D. , & Peters, D. H. (2018). Implementation research: New imperatives and opportunities in global health. Lancet Health Policy, 392(10160), 2214–2228. 10.1016/S0140-6736(18)32205-0 30314860

[14] Warren, A. M. , Frongillo, E. A. , & Rawat, R. (2020). Building implementation science in nutrition. Advances in Nutrition, 11, 1392–1398. 10.1093/advances/nmaa066 32583850 PMC7490173

[15] Jacobson, N. , Butterill, D. , & Goering, P. (2003). Development of a framework for knowledge translation: Understanding user context. Journal of Health Services Research & Policy, 8(2), 94–99. 10.1258/135581903321466067 12820671

[16] Tumilowicz, A. , Ruel, M. T. , Pelto, G. , Pelletier, D. , Monterrosa, E. C. , Lapping, K. , Kraemer, K. , De Regil, L. M. , Bergeron, G. , Arabi, M. , Neufeld, L. , & Sturke, R. , Society for Implementation Science in Nutrition . (2019). Implementation science in nutrition: Concepts and frameworks for an emerging field of science and practice. Current Developments in Nutrition, 3(3), nzy080. 10.1093/cdn/nzy080 30864563 PMC6400593

[17] Shroff, Z. , Aulakh, B. , Gilson, L. , Agyepong, I. , El‐Jardali, F. , & Ghaffar, A. (2015). Incorporating research evidence into decision‐making processes: Researcher and decision‐maker perceptions from five low‐ and middle‐income countries. Health Research Policy and Systems, 13, 1–14. 10.1186/s12961-015-0059-y 26621364 PMC4666035

[18] Allen, L. , Benoist, B. , Dary, O. , & Hurrell, R. (2006). WHO/FAO Guidelines on Food Fortification with Micronutrients.

[19] Darwar, R. , Rowe, L. A. , Chadha, M. , Sanson‐Rosas, A. M. , & Arabi, M. (2023). A blueprint for fortification planning and programming: Lessons learned from an analytical review of existing fortification frameworks. Maternal & Child Nutrition, e13571. 10.1111/mcn.13571 38155486 PMC12647979

[20] Food Fortification Initiative . (2024). Food Fortification Initiative, Resources. https://www.ffinetwork.org/tools

[21] The Society for Implementation Science in Nutrition. https://www.implementnutrition.org/

[22] M. de la S. du B. F.—and Centres de Contrôle et de Prévention des Maladies des Etats Unis d'Amérique . (2023). Enquête Nationale sur les Micronutriments au Burkina Faso, 2020. Module 2 Rapport sur les Données de l'Enquête sur les Enfants. Rapport Final, Décembre 2023. Ouagadougou, Burkina Faso.

[23] Global Alliance for Improved Nutrition . (2018). Market survey in Burkina Faso using the Fortification Assessment Coverage Toolkit (FACT), 2017. Geneva.

[24] Ministère de la Santé du Burkina Faso and Centre de Contrôle et de Prévention des Maladies des États‐Unis d'Amérique . (2022). Enquête Nationale sur les Micronutriments au Burkina Faso, 2020. Module 1: Rapport sur la Méthodologie de l'Enquête, le Données sur les Ménages et la fortification des Aliments. Ouagadougou, Burkina Faso.

[25] Institut National de la Statistique et de la Démographie (INSD) . (2022). Enquête Harmonisée sur le Conditions de Vie des Ménages 2018–2019. Burkina Faso, 2018–2019. 10.48529/d5s2-kq92

[26] Some´, J. W. , & Jones, A. D. (2018). The influence of crop production and socioeconomic factors on seasonal household dietary diversity in Burkina Faso. PLoS ONE, 13(5), e0195685. 29771910 10.1371/journal.pone.0195685 PMC5957435

[27] Bandyopadhyay, A. , Haile, B. , Azzarri, C. , & Some, J. (2021). Analyzing the drivers of household dietary diversity: Evidence from Burkina Faso. Food and Nutrition Bulletin, 42(4), 530–550. 34467801 10.1177/03795721211029092 PMC8637355

[28] Ndiaye, B. , Siekmans, K. , Kung'u, J. , & Wade, S. (2015). Distribution of iron, vitamin A and zinc deficiencies in children and women in Senegal. European Journal of Nutrition & Food Safety, 5(5), 908–909. 10.9734/EJNFS/2015/21158

[29] Agence National de la Statistique et de la Démographie (ANSD) . (2022). Enquête Harmonisée sur le Conditions de Vie des Ménages 2018–2019. Senegal, 2018–2019. Senegal. 10.48529/hhhx-j012

[30] Federal Government of Nigeria (FGoN) and the International Institute of Tropical Agriculture (IITA) . (2024). National Food Consumption and Micronutrient Survey 2021. Key Findings. FGoN and IITA.

[31] Federal Government of Nigeria (FGoN) and the International Institute of Tropical Agriculture (IITA) . (2024). National Food Consumption and Micronutrient Survey 2021. Final Report. FGoN and IITA.

[32] National Bureau of Statistics (NBS) . (2021). Living Standards Survey 2018–2019, Nigeria, 2018–2019. 10.48529/8gbe-z155

[33] Global Alliance for Improved Nutrition and Oxford Policy Management . (2018). Fortification Assessment Coverage Toolkit (FACT) survey in two Nigerian states: Ebonyi and Sokoto, 2017. Geneva: Global Alliance for Improved Nutrition.

[34] Food Fortification Initiative . (2018). Fortification Assessment Coverage Tool (FACT) survey in two Nigerian states: Kano and Lagos, 2015.

[35] Olugbenga Ben, O. , Blessing, M. O. , & Lessing, O. (2023). Progress in food fortification in Nigeria—Historical overview, current issues, consumer perceptions and awareness, and the need for additional vehicles. Food Science and Nutrition Research, 6(2), 1–17.

[36] Ojji, D. (2024). Nigeria Sodium Study (NaSS): Updates. Presentation to the Bouillon Fortification Country Working Group, June 20, 2024. Abuja, Nigeria.

[37] Kohli, N. , Pradhan, M. , & Menon, P. (2014). A network analysis of nutrition stakeholders in Odisha. International Food Policy Research Institute.

[38] (2023). WHO global report on sodium intake reduction. Geneva: World Health Organization.

[39] Adams, K. P. , Vosti, S. A. , Somé, J. S. , Tarini, A. , Bercher, E. , Koudougou, K. , & Engle‐Stone, R. (2024). Micronutrient‐fortified bouillon as a strategy to improve the micronutrient adequacy of diets in Burkina Faso. Annals of the New York Academy of Sciences, 1536, 135–150. 10.1111/nyas.15155 38809659

[40] Adams, K. P. , Vosti, S. A. , Tarini, A. , Beye, M. , Pachón, H. , Kiselova, S. , & Engle‐Stone, R. (2024). The potential contributions of bouillon fortification to meeting micronutrient requirements among women and preschool children in Senegal: A modeling study using household consumption and expenditure survey data. Annals of the New York Academy of Sciences, 1537, 98–112. 10.1111/nyas.1515617496632 38973341

[41] Adams, K. P. , Vosti, S. A. , Becher, E. , Ishaya, F. , & Engle‐Stone, R. (2024). Bouillon fortification as a strategy to address inequities in micronutrient adequacy of diets in Nigeria. Annals of the New York Academy of Sciences, 1540(1), 235–250. 10.1111/nyas.15207 39255239

[42] Thompson, L. , Becher, E. , Adams, K. P. , Haile, D. , Walker, N. , Tong, H. , Vosti, S. A. , & Engle‐Stone, R. (2024). Modeled impacts of bouillon fortification with micronutrients on child mortality in Senegal, Burkina Faso, and Nigeria. Annals of the New York Academy of Sciences, 1537, 82–97. 10.1111/nyas.15174 38922959

[43] Vosti, S. A. , Jarvis, M. , Anjorin, O. M. , Engle‐Stone, R. , Beye, M. , Ishaya, F. , Koudougo, K. , Oni, B. , Somda, H. , & Adams, K. P. (2024). The costs and the potential allocation of costs of bouillon fortification: The cases of Nigeria, Senegal, and Burkina Faso. Annals of the New York Academy of Sciences, 1541, 181–201. 10.1111/nyas.15234 39429034 PMC11580765

[44] Gouvernement du Burkina Faso . (2013). *Arrêté interministériel N°2013‐1033/MS/MASA/MICA/MEF du 27 septembre 2013 portant réglementation de l'importation, de la commercialisation et de l'utilisation du sel au Burkina Faso*.

[45] Helen Keller International, Country Working Group (CWG), and GroundWork . (2024). Salt and Sodium Intake Survey Burkina Faso 2023 (SSIS Burkina Faso 2023). Ouagadougou, Burkina Faso.

[46] Gouvernement du Sénégal . (2014). *Arrêté interministériel N° 14613 du 15‐09‐2014 modifiant les taux de conformité du sel en iode*.

[47] Helen Keller International and GroundWork . (2024). Salt and Sodium Intake Survey Senegal 2023 (SSIS Senegal 2023). Dakar, Senegal.

[48] Tarini, A. , Manger, M. S. , Brown, K. H. , Mbuya, M. N. N. , Rowe, L. A. , Grant, F. , Black, R. E. , & McDonald, C. M. (2021). Enablers and barriers of zinc fortification; Experience from 10 low‐ and middle‐income countries with mandatory large‐scale food fortification. Nutrients, 13(6), 2051. 10.3390/nu13062051 34203987 PMC8232736

[49] Lazo‐Porras, M. , Perez‐Leon, S. , Cardenas, M. K. , Pesantes, M. A. , Miranda, J. J. , Suggs, L. S. , Chappuis, F. , Perel, P. , & Beran, D. (2020). Lessons learned about co‐creation: Developing a complex intervention in rural Peru. Global Health Action, 13(1), 1754016. 10.1080/16549716.2020.1754016 32406330 PMC7269078

[50] Vargas, C. , Whelan, J. , Brimblecombe, J. , & Allender, S. (2022). Co‐creation, co‐design and co‐production for public health: A perspective on definitions and distinctions. Public Health Research & Practice, 32(2), e3222211. 10.17061/phrp3222211 35702744

[51] Lancet Commission on Strengthening the Use of Epidemiological Modelling of Emerging and Pandemic Infectious Diseases . (2024). How modelling can better support public health policy making: The Lancet Commission on Strengthening the Use of Epidemiological Modelling of Emerging and Pandemic Infectious Diseases. Lancet, 403(10429), 789–791. 38141627 10.1016/S0140-6736(23)02758-7

[52] Whelan, J. , Brimblecombe, J. , Christian, M. , Vargas, C. , Ferguson, M. , McMahon, E. , Lee, A. , Bell, C. , Boelsen‐Robinson, T. , Blake, M. R. , Lewis, M. , Alston, L. , & Allender, S. (2023). CO‐Creation and Evaluation of Food Environments to Advance Community Health (COACH). AJPM Focus, 2(3), 100111. 10.1016/j.focus.2023.100111 37790671 PMC10546519

[53] Meloncelli, N. , Young, A. , Christoffersen, A. , Rushton, A. , Zhelnov, P. , Wilkinson, S. A. , Scott, A. M. , & de Jersey, S. (2023). Co‐designing nutrition interventions with consumers: A scoping review. Journal of Human Nutrition and Dietetics, 36, 1045–1067. 10.1111/jhn.13082 36056610

[54] Even, B. , Crawford, S. , Shittu, O. F. , Lundy, M. , Wertheim‐Heck, S. , Samuel, F. O. , Talsma, E. F. , Pastori, G. , Thi Le, H. , Hernandez, R. , Brouwer, I. D. , & Béné, C. (2024). From streets to tables: Bottom–up co‐creation case studies for healthier food environments in Vietnam and Nigeria. Current Developments in Nutrition, 8(8), 104395. 10.1016/j.cdnut.2024.104395 39157008 PMC11327531

[55] Pastrana, N. A. , Beran, D. , Somerville, C. , Heller, O. , Correia, J. C. , & Suggs, L. S. (2020). The process of building the priority of neglected tropical diseases: A global policy analysis. PLOS Neglected Tropical Diseases, 14(8), e0008498. 10.1371/journal.pntd.0008498 32785262 PMC7423089

[56] Ghaffar, A. , Gupta, A. , Kampo, A. , & Swaminathan, S. (2021). The value and promise of embedded research. Health Research Policy and Systems, 19(2), 99. 10.1186/s12961-021-00744-8 34380500 PMC8356373

[57] Engle‐Stone, R. , Wessells, K. R. , Haskell, M. J. , Kumordzie, S. M. , Arnold, C. D. , Da vis, J. N. , Becher, E. R. , Fuseini, A. D. , Nyaaba, K. W. , Tan, X. , Adams, K. P. , Lietz, G. , Vosti, S. A. , & Adu‐Afarwuah, S. (2024). Effect of multiple micronutrient‐fortified bouillon on micronutrient status among women and children in the Northern Region of Ghana: Protocol for the Condiment Micronutrient Innovation Trial (CoMIT), a community‐based randomized controlled trial. PLoS ONE, 19(5), e0302968. 10.1371/journal.pone.0302968 38709803 PMC11073681

[58] Wessells, K. R. , Kumordzie, S. M. , Becher, E. , Davis, J. N. , Nyaaba, K. W. , Zyba, S. J. , Arnold, C. D. , Tan, X. , Vosti, S. A. , Adams, K. P. , Haskell, M. , Adu‐Afarwuah, S. , & Engle‐Stone, R. (2023). Acceptability of multiple micronutrient‐fortified bouillon cubes among women and their households in two districts in the Northern region of Ghana. Current Developments in Nutrition, 8(1), 102056. 38304733 10.1016/j.cdnut.2023.102056 PMC10832376

[59] Adebayo, T. (2024). Fortified bouillon cubes are seen as a way to curb malnutrition in Africa. Associated Press, World News.

[60] Scaling Community of Practice . https://scalingcommunityofpractice.com

[61] Scaling Community of Practice—Nutrition Scaling Working Group . https://scalingcommunityofpractice.com/groups/nutrition/members/

[62] Condiment Micronutrient Innovation Trial (CoMIT) Project . Institute for Global Nutrition, University of California, Davis. https://globalnutrition.ucdavis.edu/research/research_proj

[63] Micronutrient Intervention Modeling (MINIMOD) Project . Department of Agricultural and Resource Economics, University of California, Davis. https://minimod.ucdavis.edu/

[64] Damschroder, L. J. , Aron, D. C. , Keith, R. E. , Kirsh, S. R. , Alexander, J. A. , & Lowery, J. C. (2009). Fostering implementation of health services research findings into practice: a consolidated framework for advancing implementation science. Implementation Science, 4(1), 50. 19664226 10.1186/1748-5908-4-50 PMC2736161

